# Framing Health Care Instruction: An Information Literacy Handbook for the Health Sciences

**DOI:** 10.5195/jmla.2020.968

**Published:** 2020-07-01

**Authors:** Rebecca C. Hedreen

**Affiliations:** 1 hedreenr1@southernct.edu, Hilton C. Buley Library, Southern Connecticut State University, New Haven, CT

The Association of College & Research Libraries (ACRL) Information Literacy (IL) Framework replaced the ACRL Information Literacy Competency Standards for Higher Education in 2016, but many librarians are still struggling to incorporate the ACRL Framework into their library instruction. This book contains everything needed to do that work: explanations of the ACRL Framework, how it fits into health care and health sciences education, and lots of examples that are ready to be used or modified.

The first chapter maps accreditation standard components across the health fields onto the ACRL Framework. Nursing, medical, dental, pharmacy, occupational therapy, physical therapy, and veterinary standards were all identified as having at least one ACRL Framework component mentioned. This chapter will be very useful for librarians who are trying to gain a foothold into or expand library and/or IL instruction.

The following chapters are brief descriptions of the ACRL Framework components with specific importance in the health sciences (authority, creation, value, inquiry, conversation, and searching); a useful “At a Glance” graphic, written by the editors (except for chapter 5, “Inquiry in the Health Sciences,” which is written by Elizabeth Yost and Marianne Sade); and then a selection of case studies with lesson plans and exercises from a wide variety of fields, levels, and schools in the health sciences. For anyone familiar with the ACRL Framework, the descriptive portions of the chapters will be familiar. However, for those who need a refresher on the ACRL Framework or those new to health care library instruction, they will prove very useful in relating the ACRL Framework to health care. The graphics provide a good summary of the topics, including a couple of points as “Special considerations for the health sciences” for each component.

But the most useful parts of the book for librarians will likely be the case studies. With six case studies for each of the six components, there are plenty of ideas here. Nursing, medicine, public health, physical and occupational therapy, social work, veterinary medicine, and dentistry are represented, as are undergraduate-, graduate-, and professional-level work. Lessons include evaluating different types of information, the publication process and what it means to the content, and building of useful research questions and search strategies. Some of the lessons are explicitly librarian-led, while others could be incorporated into regular course work.

The index has a few quirks: public health is listed as “health, public”; undergraduate- and graduate-level work is listed under “students, undergraduate” and “students, graduate” (etc.). While most of the subjects are listed, the veterinary case study is not listed under the veterinary medicine subject, but it is indexed under the university (but not specifically as the veterinary program). While the introduction specifically mentions hospital library instruction, there is no entry for “hospital” in the index; “residents” is listed.

Overall, this is an excellent addition to any collection that serves the health sciences. Any librarian who does instruction in the health sciences will find this book valuable, but especially those who do more formal instruction sessions and those who work with faculty to incorporate information literacy into the curriculum.

